# A comparison of the effects of connective tissue massage and classical massage on chronic mechanical low back pain

**DOI:** 10.1097/MD.0000000000033516

**Published:** 2023-04-14

**Authors:** Göktuğ Er, İnci Yüksel

**Affiliations:** a Eastern Mediterranean University, Faculty of Health Sciences, Department of Physiotherapy and Rehabilitation, Famagusta, North Cyprus via Mersin, Turkey.

**Keywords:** massage, nervous system, pain, spine

## Abstract

The study aimed to compare the effects of connective tissue massage (CTM) and classical massage (CM) in patients with chronic mechanical low back pain on pain and autonomic responses and to determine the most effective manual therapy method.

**Methods:**

Seventy individuals with chronic mechanical low back pain were randomly divided into CTM (n = 35) and CM (n = 35) groups. The participants were given a 4-week treatment protocol consisting of a hot pack, exercise, and CTM or CM for 20 sessions. A visual analog scale was used to measure pain intensity. Heart rate, blood pressure, and skin temperature were measured for the evaluation of autonomic responses. In addition, disability (Oswestry disability index), quality of life (short form 36), and sleep quality (Pittsburgh sleep quality index) were evaluated. Participants were assessed before and after the 4-week treatment period as well as at the end of the 6-week follow-up period. In addition, visual analog index measurements were repeated at the end of each treatment week.

**Results:**

Pain intensity was decreased in both groups (*P* < .05). However, CM was more effective than CTM at the end of the 2^nd^ week (*P* < .05). In autonomic responses results, there were increases in peripheral skin temperatures in both groups (*P* < .05). Disability, quality of life, and sleep quality improved in both groups (*P* < .05). There were no differences between the groups relating to autonomic responses, disability, quality of life, and sleep quality (*P* > .05).

**Conclusion:**

The results of this study showed that massages were similar effect. The fact that CM is a frequently used technique in pain management and is as effective as CTM in autonomic responses will make it more preferred in the clinic.

## 1. Introduction

Mechanical low back pain is defined as a condition that causes tension, pain, or stiffness in the lumbar region without any specific cause (e.g., infection, tumor, osteoporosis, fracture, etc).^[1,2]^ Mechanical low back pain has become one of the biggest problems for public health systems in the Western world and continues to be a major health problem in today’s world.^[1]^ A review conducted in 54 countries revealed that the disease prevalence was 18.3%.^[3]^

Chronic low back pain negatively affects daily life and causes other problems in addition to pain. Disability due to pain can severely limit participation in work, social, and family life.^[3]^ Causative factors in most individuals are overuse, stress from repetitive movement, and/or hypermobility. In some cases, a reduction in stress (due to immobilization or hypomobility) may be the causative factor.^[4]^ These conditions cause negative changes in the connective tissue architecture.^[4]^

Another common finding of low back pain are neurophysiological changes caused by autonomic nervous system imbalance.^[5]^ Autonomic dysfunction presents itself as variability in heart rate and body temperature and elevated blood pressure.^[6,7]^ It has been reported that heart rate variability may result from disability, pain, and distress in low back pain, which will result in increased sympathetic system activity.^[8]^ El-Badawy et al^[9]^ demonstrated a sympathetic dysfunction in individuals with chronic low back pain using electrophysiological methods. The evaluation of the autonomic nervous system function is often overlooked in clinics.

Pharmacotherapy is widely used for chronic low back pain and other accompanying problems. However, the possibility of drug abuse and adverse effects has led researchers to focus on non-pharmacological approaches.^[3,10]^ The side effects of long-term opioid use were reviewed in a study.^[11]^ More than half of the patients who received opioid treatment for at least 3 months have been shown to continue taking drugs even years after treatment. The prolonged use of these drugs results in opioid dependence. Dependence is divided into physical (compensatory adaptations in parts of the brain that control somatic functions), psychological (suppressing unpleasant emotional effects, including anhedonia, dysphoria, etc), and experimental. The withdrawal of opioids causes agitation, insomnia, diarrhea, rhinorrhea, piloerection, and hyperalgesia.

However, massage is a safe treatment option with few side effects in patients with low back pain. It is an effective therapy in some types of headaches, muscle soreness, and mechanical neck pain.^[12,13]^ These positive features ensure that massage is included in low back pain treatment guidelines.^[14–16]^ The classical massage (CM)–also known as “Swedish massage”–provides a symptomatic reduction in pain, especially in musculoskeletal diseases. CM improves physiological and clinical outcomes by increasing the pain threshold with the release of endorphins.^[17]^

Connective tissue massage (CTM) has local and reflex effects that reshape the tissue through manipulation. Influences of the CTM stimuli in the skin and subcutaneous fascial layers result in reflex effects on the autonomic nervous system.^[6,18]^ It was claimed that sympathetic activity decreases with the cutaneous-visceral reflexes after applying CTM.^[17,19,20]^ As a result of the effects of CTM on the autonomic nervous system, blood circulation and mobility increase, pain reduces, and sleep quality improves.^[6,18,21]^

Previous studies have stated that CM and CTM reduced pain and disability and increased sleep quality and quality of life in patients with fibromyalgia, chronic neck pain, and chronic low back pain.^[22–24]^ Because of these effects, both massages have been widely used in clinical practice. However, it is unknown which form is more effective in patients with mechanical low back pain.^[7,25]^

Based on the information discussed above, the primary aim of the study was to determine the most effective manual therapy by comparing the effects of CM and CTM on pain in patients with chronic mechanical low back pain (CMLBP). The second aim was to compare the effects of massage methods on autonomic responses, disability, quality of life, and sleep quality.

## 2. Methods

### 2.1. Study design

This study was designed as a randomized, comparative, and mono-center study. The study was conducted in the Orthopedic Rehabilitation Unit of the Health Sciences Faculty of Eastern Mediterranean University between March 2019 and August 2021. The study was approved by the Health Ethics Committee (ETK00-2019-0177) and registered into the Clinical Trials (NCT04211701). This study was conducted in accordance with the rules of the Declaration of Helsinki.

All assessments and treatments were conducted by the same physiotherapist (GE) to standardize the study. The physiotherapist had 7 years of experience with the subject.

### 2.2. Participants

Patients diagnosed with chronic mechanical low back pain by orthopedics and traumatology doctors in local state hospital were included in the study. The G*Power software program (version 3.0; Kiel, Germany) was used to determine the required sample size for the study. Thus, a sample consisting of 54 participants (27 per group) was needed to obtain 80% power with Cohen effect size of d = .80 (for 80% power), α = .05, and β = .20. It was predicted that some participants would not be able to fulfill the treatment protocols for various reasons, and the sample sizes of each group were increased by 30%.

Before the enrollment process, the participants were informed about the study and signed informed consent forms were obtained. Thus, 76 interviewed participants who complied with the inclusion and exclusion criteria.

#### 1.2.2. Inclusion and exclusion criteria

The inclusion criteria were as follows: aged between 20 and 60 years, referred to a physiotherapy clinic, the presence of mechanical low back pain for more than 12 weeks, the absence of neurological problems originating from the lumbar region, and not participating in any physiotherapy and rehabilitation program in the last 6 months.

The exclusion criteria were as follows: the presence of axial spondyloarthropathy, pain and numbness radiating to the hip and lower extremities, congenital malalignment in columna vertebralis, malignity, lower extremity inequality greater than 1 cm, a difference in blood pressure measurements of more than 10 mm Hg between the 2 arms, and/or pregnancy.

### 2.3. Randomization

**Figure 1.**
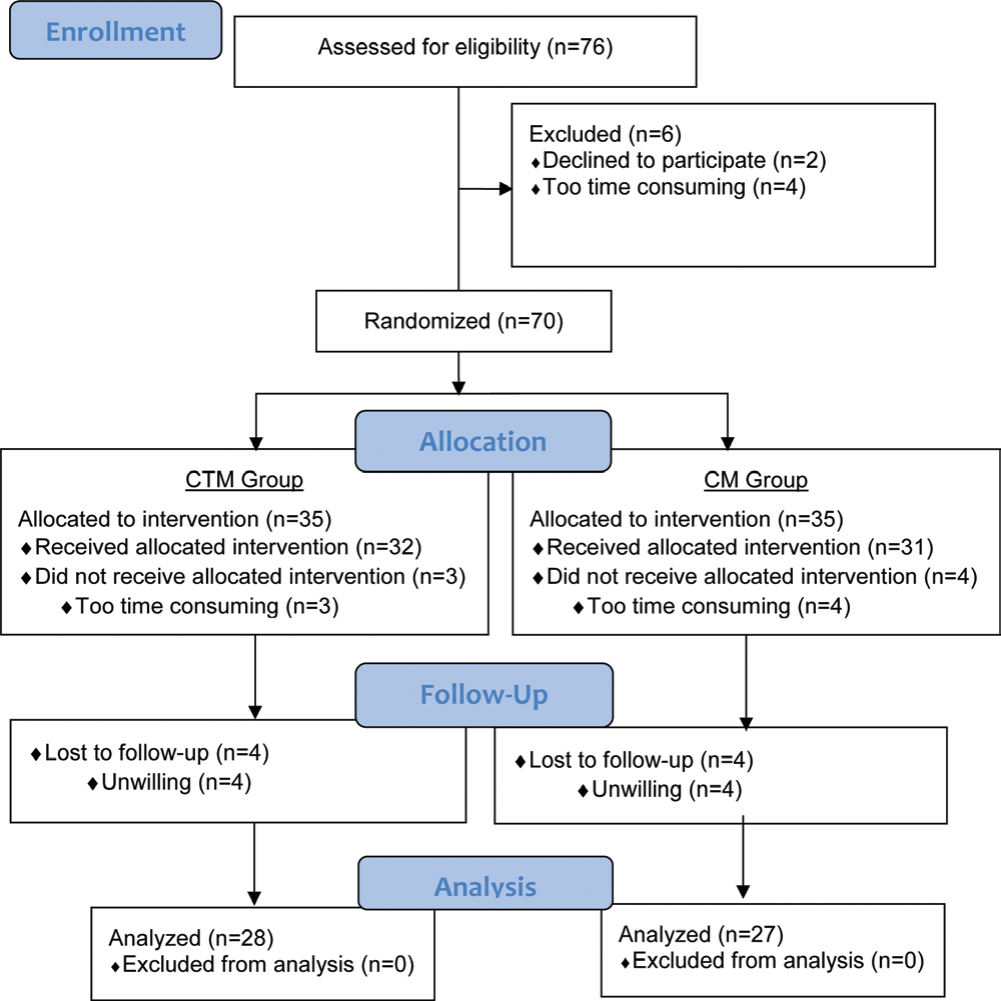
The participant flow chart according to CONSORT is displayed in Figure 1.

The patients were randomly assigned to one of the following 2 groups: CTM group (n = 35) and CM group (n = 35). Using the minimization method, the groups were made similar in terms of gender, age, and occupation. The participant flow chart is displayed in Figure 1.

### 2.4. Interventions

Patients were treated with CM or CTM after a hot pack application (20 minutes) to the lumbar region. In addition, all the participants were given lumbar strengthening and stretching exercises (Table 1). Both groups were treated 5 days a week, for a total of 20 sessions.

**Table 1:** Exercise program.

Lumbar extensor stretching while in supine position (hold in 20 sec, 5 reps)
2) Posterior pelvic tilt (10 reps)
3) Shoulder bridging (10 reps)
4) Cat–Camel exercise (10 reps)
5) Hip twist exercise (grade 1, 10 reps)
6) Clam exercise (grade 1, 10 reps)
7) Hamstring stretch by straight leg raise (hold in 20 sec, 5 reps)

#### 1.2.4. Connective tissue massage

The participant was seated on a stool with the entire back and sacral region open, hip and knee flexed at 90°, and feet on the ground during the treatment. While sitting, it was ensured that the back of the individual was straight and their hands supported the thighs for slight loosening of the connective tissue. CTM was applied by the physiotherapist in the form of short and long pulling strokes in a hooking style with the third finger flexed at 45° to 60° from the distal phalanx. CTM was applied to the basic region during the first 3 sessions and then progressed to the lower thoracic region, scapular region, and interscapular region, for the following sessions respectively. CTM pulls were performed 3 to 4 times for each area. Decreased tension, the appearance of lines of hyperemia, and appropriate vascular response were identified as the criteria for progression to other regions. CTM application initially lasted for 5 minutes and reached 20 to 25 minutes with the progression of the massage to the upper segments.

#### 2.2.4. Classical massage

CM was applied to the lower and upper back regions in the prone position. Massage oil was used as an intermediate. After general stroking to the entire back, stroking and kneading were applied to the erector spine, latissimus dorsi, and gluteus maximus muscles in the lower back. An upper back massage was performed after the lower back massage and stroking and kneading were applied to the erector spine and trapezius muscles. The back massage ended with a general stroking application. The duration of the massage was approximately 15 to 20 minutes.

### 2.5. Outcome measures

All the outcomes were measured by the same physiotherapist the day before the first treatment session, the day after the completion of all treatments, and at the end of the 6-week follow-up. In addition, pain intensity was measured at the end of the 5^th^, 10^th^, and 15^th^ sessions.

#### 1.2.5. Primary outcome measure

##### 1.2.5.1. Pain intensity

A visual analog scale (VAS) was used to measure pain intensity.^[26]^ Participants marked the intensity of pain they felt on a 10-cm line, where 0 indicated no pain and 10 represented very severe pain.

#### 2.2.5. Secondary outcome measures

##### 2.1.2.5. Autonomic functions

Blood pressure, heart rate, and peripheral and local skin temperature changes were measured to evaluate the possible effects of CM and CTM on the autonomic responses.

The blood pressure measurements were taken using a Erka Switch model manual sphygmomanometer. For the measurement, the participants were seated in a chair with back and arm support with their feet on the ground. After resting in this position for 5 minutes, the forearm was fixed on a flat table and the arm was brought to the level of the right atrium. If the individual smoked, drank coffee, or exercised, measurements were performed 30 minutes later.^[27,28]^ Heart rate was measured using a Zondan A2 model pulse oximeter attached to the fingertip in the sitting position.

Peripheral skin temperature was measured from the medial longitudinal arch, which was reported to be the hottest point of the foot.^[29]^ For the measurements, the laboratory was set at a temperature of 21°C to 25°C with no direct sunlight. Individuals were asked to take off their shoes and socks and lie down in a supine position on the treatment table. A 15-minute waiting time was required for the moisture and heat from shoes and socks to disappear and for the foot to return to its normal temperature.^[29,30]^ Measurements were taken using a Testo 830-T1 model laser marking heat meter. The device had a sensitivity of 0.1°C resolution.

Local skin temperatures were evaluated in the same laboratory conditions prepared for the peripheral skin temperature. Temperature measurements were performed bilaterally from the following anatomical points stated by Holey et al^[7]^: Inferior costal margins right and left, the highest point of iliac crest (superior side), and posterior superior iliac spine (inferior side).

### 2.6. Disability

The Oswestry disability index (ODI) was used to evaluate the disability of the individuals.^[31]^ The index had 10 items, which were scored on a scale of 0 to 5 points based on functional performance, with higher scores indicating more severe disabilities.

### 2.7. Health-related quality of life

The short form 36 (SF-36) was used to assess health-related quality of life.^[32]^ The questionnaire consisted of 36 questions that evaluated the quality of life of individuals under eight main headings. The survey score ranged between 0 and 100. High scores indicated good health.

### 2.8. Sleep quality

The Pittsburgh sleep quality index (PSQI) was used to determine sleep quality. PSQI is the preferred index in clinical and research studies.^[33]^ PSQI determines sleep quality and had 19 self-reported questions. The questions were scored between 0 and 3 points using a 4-point Likert scale. The index had 7 subcategories. Scores from the subcategories formed the total score. High scores indicated poor sleep quality.

### 2.9. Statistical analysis

The obtained data were evaluated with the SPSS Version 22.0 program (SPSS Inc., Chicago, IL). The level of significance was accepted as *P* < .05. All data were tested for normality (Shapiro–Wilk test). The data were not suitable for normal distribution. The chi-square test was used for comparisons of demographic information’s. The Mann–Whitney *U* test was used to determine the differences between the groups, and Friedman analysis was used to compare the data measured at different times. The post hoc Dunn test was applied to intra group analysis, aiming to prevent type-1 errors.

## 3. Results

The demographic information’s of the groups were similar (Table 2).

**Table 2:** Comparisons of demographic information of individuals.

	CTM (n = 28) (X ± SD)	CM (n = 27) (X ± SD)	*P* value *
Age (yr)	36.86 ± 12.63	37.09 ± 11.59	.86
Height (m)	1.68 ± 0.09	1.72 ± 0.09	.15
Weight (kg)	72.26 ± 16.06	77.69 ± 11.76	.08
BMI (kg/m^2^)	25.29 ± 4.37	26.34 ± 3.23	.22
	n	n	*P* value†
Gender			
Male	14	13	.99
Female	14	14	
Alcohol use (Yes)	6	5	.99
Smoking (Yes)	12	14	.81
Occupation			
White collar	7	8	.62
Blue collar	10	8	
Student	8	9	
House wife	3	2	

BMI = body mass index, CM = classical massage, CTM = connective tissue massage.

* Mann–Whitney *U* test.

† Chi-square test.

### 3.1. Pain intensity

When the time-dependent changes in the intensity of pain were analyzed, it was found that both treatments provided significant improvements (Table 3). There were statistically significant decreases in both groups posttreatment and at the end of the 6-week follow-up VAS scores compared to pretreatment (posttreatment *P* < .001 and at the end of the 6-week follow-up *P* < .001 in CTM group; posttreatment *P* < .001 and at the end of the 6-week follow-up *P* < .001 in CM group) (Table 4). The effects of the treatments lasted for a long time.

**Table 3:** Comparison of pain intensity of the groups.

Variable	Measurement Times	CTM (n = 28) X ± SD	CM (n = 27) X ± SD	*P* value*
VAS (cm)	Pretest	5.52 ± 2.03	5.43 ± 1.90	.84
	1^st^ wk	3.76 ± 2.27	3.22 ± 1.64	.35
	2^nd^ wk	2.60 ± 1.65	1.90 ± 1.71	**.04**
	3^rd^ wk	1.48 ± 1.42	1.10 ± 1.13	.34
	Posttest	0.85 ± 1.28	0.38 ± 0.44	.55
	Follow-up	1.00 ± 1.08	0.82 ± 1.09	.46
	*P* value†	**<.001**	**<.001**	-

Bold indicates significant values.

CM = classical massage, CTM = connective tissue massage, VAS = visual analog scale.

* Mann–Whitney *U* test.

† Friedman test.

**Table 4:** Change of pain intensity of the groups according to time.

Variables	Measurement Times	CTM (n = 28)		CM (n = 27)	
		X ± SD	*P* value*	X ± SD	*P* value*
VAS (cm)	Pretest	5.52 ± 2.03	.06	5.43 ± 1.90	.39
	1^st^ wk	3.76 ± 2.27		3.22 ± 1.64	
	1^st^ wk	3.76 ± 2.27	.94	3.22 ± 1.64	.43
	2^nd^ wk	2.60 ± 1.65		1.90 ± 1.71	
	2^nd^ wk	2.60 ± 1.65	.94	1.90 ± 1.71	.99
	3^rd^ wk	1.48 ± 1.42		1.10 ± 1.13	
	3^rd^ wk	1.48 ± 1.42	.74	1.10 ± 1.13	.47
	Posttest	0.85 ± 1.28		0.38 ± 0.44	
	Pretest	5.52 ± 2.03	**<.001**	5.43 ± 1.90	**<.001**
	Posttest	0.85 ± 1.28		0.38 ± 0.44	
	Posttest	0.85 ± 1.28	.99	0.38 ± 0.44	.99
	Follow-up	1.00 ± 1.08		0.82 ± 1.09	
	Pretest	5.52 ± 2.03	**<.001**	5.43 ± 1.90	**<.001**
	Follow-up	1,00 ± 1.08		0.82 ± 1.09	

Bold indicates significant values.

CM = classical massage, CTM = connective tissue massage, VAS = visual analog scale.

* Post hoc Dunn test.

When the pretreatment, 1^st^ week, 3^rd^ week, posttreatment, and at the end of the 6-week follow-up VAS scores were compared between the 2 groups, no difference was found (*P* > .05) (Table 3). However, pain relief in the 2^nd^ week of the treatment of the CM group was significantly higher than that of the CTM group (*P* = .04) (Table 3).

### 3.2. Autonomic functions

When the time-dependent changes in the autonomic functions were analyzed, it was found that both treatments provided significant increases in right peripheral temperatures. Besides, it was found that there was a significant increase in left peripheral temperature in the CTM group (Table 5). There were statistically significant increases in the CTM group at the end of the 6-week follow-up both peripheral temperatures compared to pretreatment (*P* = .01 in left peripheral; *P* = .03 in right peripheral) (Table 6). There was a statistically significant increase in the CM group posttreatment right peripheral temperature compared to pretreatment (*P* = .04) (Table 6).

**Table 5:** Comparisons of autonomic functions of groups.

Variables	Measurement Times	CTM (n = 28) X ± SD	CM (n = 27) X ± SD	*P* value*
Heart rate (BPM)	Pretest	75.77 ± 10.08	78.31 ± 11.31	.26
	Posttest	75.73 ± 8.53	77.63 ± 9.81	.33
	Follow-up	78.37 ± 7.63	80.85 ± 9.34	.26
	*P* value†	.06	.10	-
Systolic blood pressure (mm Hg)	Pretest	110.26 ± 18.47	108.06 ± 14.16	.72
	Posttest	108.77 ± 16.61	109.59 ± 14.06	.67
	Follow-up	108.20 ± 16.02	110.62 ± 15.07	.49
	*P* value†	.93	.26	-
Diastolic blood pressure (mm Hg)	Pretest	69.91 ± 12.99	68.23 ± 11.38	.72
	Posttest	68.77 ± 11.10	68.30 ± 10.02	.80
	Follow-up	68.50 ± 12.33	67.96 ± 10.57	.31
	*P* value†	.99	.62	-
Temperature Left inferior costal margin (°C)	Pretest	32.26 ± 1.35	31.70 ± 1.65	.17
	Posttest	32.19 ± 1.59	32.34 ± 1.52	.12
	Follow-up	32.33 ± 1.70	30.94 ± 6.30	.44
	*P* value†	.35	.12	-
Temperature Right inferior costal margin (°C)	Pretest	32.24 ± 1.38	31.18 ± 1.72	.42
	Posttest	32.16 ± 1.49	32.31 ± 1.64	.64
	Follow-up	32.45 ± 1.41	30.99 ± 6.29	.21
	*P* value†	.11	.42	-
Temperature Left iliac crest (°C)	Pretest	32.04 ± 1.56	31.78 ± 1.66	.52
	Posttest	32.10 ± 1.70	32.09 ± 1.43	.90
	Follow-up	32.22 ± 1.51	30.77 ± 6.27	.24
	*P* value†	.58	.40	-
Temperature Right iliac crest (°C)	Pretest	31.88 ± 1.64	31.80 ± 1.89	.95
	Posttest	32.15 ± 1.71	32.05 ± 1.54	.96
	Follow-up	32.28 ± 1.69	30.56 ± 6.23	.06
	*P* value†	.06	.82	-
Temperature Left posterior superior iliac spine (°C)	Pretest	33.13 ± 1.02	33.07 ± 1.26	.95
	Posttest	33.10 ± 1.08	33.40 ± 1.13	.27
	Follow-up	33.12 ± 1.33	33.10 ± 0.98	.72
	*P* value†	.30	.43	-
Temperature Right posterior superior iliac spine (°C)	Pretest	33.16 ± 1.01	33.13 ± 1.13	.88
	Posttest	33.10 ± 1.06	33.50 ± 1.06	.18
	Follow-up	33.22 ± 1.26	33.10 ± 0.91	.44
	*P* value†	.84	.13	-
Temperature Left peripheral (°C)	Pretest	29.97 ± 1.59	30.20 ± 1.82	.47
	Posttest	30.53 ± 1.77	30.80 ± 1.81	.54
	Follow-up	30.98 ± 1.53	30.72 ± 1.96	.54
	*P* value†	**.01**	.07	-
Temperature Right peripheral (°C)	Pretest	30.10 ± 1.60	30.24 ± 1.94	.87
	Posttest	30.57 ± 1.77	30.95 ± 1.68	.43
	Follow-up	31.20 ± 1.49	30.72 ± 2.00	.29
	*P* value†	**.03**	**.04**	-

Bold indicates significant values.

CM = classical massage, CTM = connective tissue massage.

* Mann–Whitney *U* test.

† Friedman test.

**Table 6:** Change of autonomic functions of groups according to time.

Variables	Measurement Times	CTM (n = 28)		CM (n = 27)	
		X ± SD	*P* value *	X ± SD	*P* value*
Temperature left peripheral (°C)	Pretest	29.97 ± 1.59	.28	30.20 ± 1.82	.99
	Posttest	30.53 ± 1.77		30.80 ± 1.81	
	Posttest	30.53 ± 1.77	.41	30.80 ± 1.81	.99
	Follow-up	30.98 ± 1.53		30.72 ± 1.96	
	Pretest	29.97 ± 1.59	**.01**	30.20 ± 1.82	.99
	Follow-up	30.98 ± 1.53		30.72 ± 1.96	
Temperature right peripheral (°C)	Pretest	30.10 ± 1.60	.28	30.24 ± 1.94	**.04**
	Posttest	30.57 ± 1.77		30.95 ± 1.68	
	Posttest	30.57 ± 1.77	.99	30.95 ± 1.68	.99
	Follow-up	31.20 ± 1.49		30.72 ± 2.00	
	Pretest	30.10 ± 1.60	**.03**	30.24 ± 1.94	.25
	Follow-up	31.20 ± 1.49		30.72 ± 2.00	

Bold indicates significant values.

CM = classical massage, CTM = connective tissue massage.

* Post hoc Dunn Test.

When the heart rate, blood pressure, and temperature measurements were compared between the 2 groups, no difference was found (*P* > .05) (Table 5).

### 3.3. Disability

When the time-dependent changes in the disability were analyzed, it was found that both treatments provided significant improvements (Table 7). There were statistically significant decreases in both groups posttreatment and at the end of the 6-week follow-up ODI scores compared to pretreatment (posttreatment *P* < .001 and at the end of the 6-week follow-up *P* < .001 in CTM group; posttreatment *P* < .001 and at the end of the 6-week follow-up *P* < .001 in CM group) (Table 8).

**Table 7:** Comparisons of disability, sleep quality, and health-related quality of life.

Variables	Measurement Times	CTM (n = 28) X ± SD	CM (n = 27) X ± SD	*P* value*
Oswestry disability index	Pretest	35.00 ± 15.47	34.09 ± 11.16	.95
	Posttest	16.36 ± 12.12	14.63 ± 11.98	.65
	Follow-up	16.57 ± 10.03	13.23 ± 8.56	.31
	*P* value†	**<.001**	**<.001**	-
PSQI subjective sleep quality	Pretest	1.54 ± 1.65	1.29 ± 0.75	.57
	Posttest	0.87 ± 0.51	1.11 ± 0.75	.19
	Follow-up	1.03 ± 0.61	1.12 ± 0.43	.41
	*P* value†	**.01**	.38	-
PSQI Sleep latency	Pretest	1.29 ± 0.96	1.17 ± 0.92	.57
	Posttest	0.63 ± 0.85	0.85 ± 0.95	.36
	Follow-up	0.80 ± 0.81	1.00 ± 0.85	.35
	*P* value†	**.01**	.21	-
PSQI sleep duration	Pretest	1.09 ± 0.95	1.20 ± 1.13	.80
	Posttest	0.57 ± 0.86	1.00 ± 0.96	.06
	Follow-up	0.73 ± 0.94	0.96 ± 0.92	.28
	*P* value†	**.03**	**.04**	-
PSQI sleep efficiency	Pretest	0.60 ± 0.88	0.69 ± 1.05	.98
	Posttest	0.20 ± 0.41	0.37 ± 0.69	.47
	Follow-up	0.13 ± 0.35	0.12 ± 0.33	.84
	*P* value†	**.03**	.11	-
PSQI sleep disturbance	Pretest	1.37 ± 0.55	1.51 ± 0.51	.19
	Posttest	1.07 ± 0.58	1.26 ± 0.59	.22
	Follow-up	1.00 ± 0.53	1.15 ± 0.54	.28
	*P* value†	**.01**	**.01**	-
PSQI sleep medication	Pretest	0.00 ± 0.00	0.00 ± 0.00	NT
	Posttest	0.00 ± 0.00	0.00 ± 0.00	NT
	Follow-up	0.00 ± 0.00	0.00 ± 0.00	NT
	*P* value†	NT	NT	-
PSQI daytime sleep dysfunction	Pretest	1.00 ± 0.91	0.83 ± 0.75	.50
	Posttest	0.47 ± 0.82	0.52 ± 0.80	.76
	Follow-up	0.60 ± 0.81	0.42 ± 0.70	.37
	*P* value†	**<.001**	**.01**	-
PSQI global score	Pretest	6.60 ± 3.12	6.69 ± 3.43	.86
	Posttest	3.80 ± 2.19	4.89 ± 3.40	.26
	Follow-up	4.03 ± 2.22	4.77 ± 2.16	.10
	*P* value†	**<.001**	**<.001**	-
SF-36 physical functioning	Pretest	66.86 ± 17.49	64.71 ± 18.86	.58
	Posttest	82.17 ± 17.30	80.56 ± 15.21	.49
	Follow-up	83.00 ± 17.05	84.04 ± 13.49	.97
	*P* value†	**<.001**	**<.001**	-
SF-36 social functioning	Pretest	66.79 ± 23.28	67.86 ± 22.74	.79
	Posttest	84.58 ± 21.94	82.87 ± 18.39	.34
	Follow-up	85.00 ± 17.18	84.13 ± 16.42	.76
	*P* value†	**<.001**	**.01**	-
SF-36 role limitations due to physical health	Pretest	33.57 ± 40.19	40.00 ± 38.92	.40
	Posttest	81.67 ± 27.02	80.56 ± 29.69	.92
	Follow-up	72.83 ± 30.28	75.96 ± 32.00	.61
	*P* value†	**<.001**	**<.001**	-
SF-36 role limitations due to emotional problems	Pretest	52.32 ± 40.69	53.33 ± 42.94	.88
	Posttest	81.11 ± 33.54	75.31 ± 34.09	.34
	Follow-up	83.33 ± 31.26	71.79 ± 39.66	.28
	*P* value†	**<.001**	.33	-
SF-36 energy/fatigue	Pretest	53.71 ± 19.68	54.71 ± 19.02	.56
	Posttest	70.33 ± 18.10	66.48 ± 14.53	.21
	Follow-up	69.50 ± 17.29	67.88 ± 11.42	.42
	*P* value†	**<.001**	**.02**	-
SF-36 emotional well-being	Pretest	69.03 ± 13.94	68.91 ± 21.03	.48
	Posttest	77.60 ± 15.50	70.37 ± 19.22	.15
	Follow-up	80.27 ± 14.40	72.62 ± 11.41	**.03**
	*P* value†	**.01**	.51	-
SF-36 pain	Pretest	58.14 ± 23.92	54.36 ± 19.50	.48
	Posttest	79.92 ± 14.20	77.96 ± 14.77	.72
	Follow-up	79.42 ± 12.54	78.94 ± 14.07	.74
	*P* value†	**<.001**	**<.001**	-
SF-36 general health	Pretest	59.86 ± 18.17	63.86 ± 11.83	.45
	Posttest	76.50 ± 15.15	75.00 ± 15.19	.63
	Follow-up	73.17 ± 16.48	75.58 ± 13.51	.78
	*P* value†	**<.001**	**<.001**	-

Bold indicates significant values.

CM = classical massage, CTM = connective tissue massage, NT = not tested, PSQI = pittsburgh sleep quality index, SF-36 = short form 36.

* Mann–Whitney *U* test.

† Friedman test.

**Table 8:** Change of disability, sleep quality, and quality of life of the groups according to time.

Variables	Measurement times	CTM (n = 28)		CM (n = 27)	
		X ± SD	*P* value*	X ± SD	*P* value*
Oswestry disability index	Pretest	35.00 ± 15.47	**<.001**	34.09 ± 11.16	**<.001**
	Posttest	16.36 ± 12.12		14.63 ± 11.98	
	Posttest	16.36 ± 12.12	.99	14.63 ± 11.98	.99
	Follow-up	16.57 ± 10.03		13.23 ± 8.56	
	Pretest	35.00 ± 15.47	**<.001**	34.09 ± 11.16	**<.001**
	Follow-up	16.57 ± 10.03		13.23 ± 8.56	
PSQI subjective sleep quality	Pretest	1.54 ± 1.65	.06	1.29 ± 0.75	.38
	Posttest	0.87 ± 0.51		1.11 ± 0.75	
	Posttest	0.87 ± 0.51	.99	1.11 ± 0.75	.57
	Follow-up	1.03 ± 0.61		1.12 ± 0.43	
	Pretest	1.54 ± 1.65	.41	1.29 ± 0.75	.28
	Follow-up	1.03 ± 0.61		1.12 ± 0.43	
PSQI sleep latency	Pretest	1.29 ± 0.96	**.04**	1.17 ± 0.92	.73
	Posttest	0.63 ± 0.85		0.85 ± 0.95	
	Posttest	0.63 ± 0.85	.99	0.85 ± 0.95	.57
	Follow-up	0.80 ± 0.81		1.00 ± 0.85	
	Pretest	1.29 ± 0.96	.24	1.17 ± 0.92	.07
	Follow-up	0.80 ± 0.81		1.00 ± 0.85	
PSQI sleep duration	Pretest	1.09 ± 0.95	.18	1.20 ± 1.13	.33
	Posttest	0.57 ± 0.86		1.00 ± 0.96	
	Posttest	0.57 ± 0.86	.99	1.00 ± 0.96	.99
	Follow-up	0.73 ± 0.94		0.96 ± 0.92	
	Pretest	1.09 ± 0.95	.47	1.20 ± 1.13	.38
	Follow-up	0.73 ± 0.94		0.96 ± 0.92	
PSQI sleep efficiency	Pretest	0.60 ± 0.88	.59	0.69 ± 1.05	.68
	Posttest	0.20 ± 0.41		0.37 ± 0.69	
	Posttest	0.20 ± 0.41	.99	0.37 ± 0.69	.08
	Follow-up	0.13 ± 0.35		0.12 ± 0.33	
	Pretest	0.60 ± 0.88	.32	0.69 ± 1.05	.28
	Follow-up	0.13 ± 0.35		0.12 ± 0.33	
PSQI sleep disturbance	Pretest	1.37 ± 0.55	.41	1.51 ± 0.51	.50
	Posttest	1.07 ± 0.58		1.26 ± 0.59	
	Posttest	1.07 ± 0.58	.99	1.26 ± 0.59	.99
	Follow-up	1.00 ± 0.53		1.15 ± 0.54	
	Pretest	1.37 ± 0.55	.14	1.51 ± 0.51	.25
	Follow-up	1.00 ± 0.53		1.15 ± 0.54	
PSQI daytime sleep dysfunction	Pretest	1.00 ± 0.91	**.02**	0.83 ± 0.75	.33
	Posttest	0.47 ± 0.82		0.52 ± 0.80	
	Posttest	0.47 ± 0.82	.99	0.52 ± 0.80	.99
	Follow-up	0.60 ± 0.81		0.42 ± 0.70	
	Pretest	1.00 ± 0.91	.18	0.83 ± 0.75	.25
	Follow-up	0.60 ± 0.81		0.42 ± 0.70	
PSQI global score	Pretest	6.60 ± 3.12	**<.001**	6.69 ± 3.43	**.01**
	Posttest	3.80 ± 2.19		4.89 ± 3.40	
	Posttest	3.80 ± 2.19	.66	4.89 ± 3.40	.99
	Follow-up	4.03 ± 2.22		4.77 ± 2.16	
	Pretest	6.60 ± 3.12	**.02**	6.69 ± 3.43	**.02**
	Follow-up	4.03 ± 2.22		4.77 ± 2.16	
SF-36 physical functioning	Pretest	66.86 ± 17.49	**<.001**	64.71 ± 18.86	**.01**
	Posttest	82.17 ± 17.30		80.56 ± 15.21	
	Posttest	82.17 ± 17.30	.99	80.56 ± 15.21	.56
	Follow-up	83.00 ± 17.05		84.04 ± 13.49	
	Pretest	66.86 ± 17.49	**<.001**	64.71 ± 18.86	**<.001**
	Follow-up	83.00 ± 17.05		84.04 ± 13.49	
SF-36 social functioning	Pretest	66.79 ± 23.28	**.01**	67.86 ± 22.74	.07
	Posttest	84.58 ± 21.94		82.87 ± 18.39	
	Posttest	84.58 ± 21.94	.99	82.87 ± 18.39	.99
	Follow-up	85.00 ± 17.18		84.13 ± 16.42	
	Pretest	66.79 ± 23.28	**.01**	67.86 ± 22.74	**.04**
	Follow-up	85.00 ± 17.18		84.13 ± 16.42	
SF-36 role limitations due to physical health	Pretest	33.57 ± 40.19	**<.001**	40.00 ± 38.92	**.01**
	Posttest	81.67 ± 27.02		80.56 ± 29.69	
	Posttest	81.67 ± 27.02	.99	80.56 ± 29.69	.99
	Follow-up	72.83 ± 30.28		75.96 ± 32.00	
	Pretest	33.57 ± 40.19	**.01**	40.00 ± 38.92	**.03**
	Follow-up	72.83 ± 30.28		75.96 ± 32.00	
SF-36 role limitations due to emotional problems	Pretest	52.32 ± 40.69	.06	53.33 ± 42.94	.37
	Posttest	81.11 ± 33.54		75.31 ± 34.09	
	Posttest	81.11 ± 33.54	.99	75.31 ± 34.09	.95
	Follow-up	83.33 ± 31.26		71.79 ± 39.66	
	Pretest	52.32 ± 40.69	**.04**	53.33 ± 42.94	.09
	Follow-up	83.33 ± 31.26		71.79 ± 39.66	
SF-36 energy/fatigue	Pretest	53.71 ± 19.68	**<.001**	54.71 ± 19.02	**.04**
	Posttest	70.33 ± 18.10		66.48 ± 14.53	
	Posttest	70.33 ± 18.10	.74	66.48 ± 14.53	.99
	Follow-up	69.50 ± 17.29		67.88 ± 11.42	
	Pretest	53.71 ± 19.68	**.01**	54.71 ± 19.02	.06
	Follow-up	69.50 ± 17.29		67.88 ± 11.42	
SF-36 emotional well-being	Pretest	69.03 ± 13.94	**.04**	68.91 ± 21.03	.25
	Posttest	77.60 ± 15.50		70.37 ± 19.22	
	Posttest	77.60 ± 15.50	.99	70.37 ± 19.22	.85
	Follow-up	80.27 ± 14.40		72.62 ± 11.41	
	Pretest	69.03 ± 13.94	**.01**	68.91 ± 21.03	.10
	Follow-up	80.27 ± 14.40		72.62 ± 11.41	
SF-36 pain	Pretest	58.14 ± 23.92	**<.001**	54.36 ± 19.50	**<.001**
	Posttest	79.92 ± 14.20		77.96 ± 14.77	
	Posttest	79.92 ± 14.20	.99	77.96 ± 14.77	.99
	Follow-up	79.42 ± 12.54		78.94 ± 14.07	
	Pretest	58.14 ± 23.92	**.01**	54.36 ± 19.50	**<.001**
	Follow-up	79.42 ± 12.54		78.94 ± 14.07	
SF-36 general health	Pretest	59.86 ± 18.17	**<.001**	63.86 ± 11.83	**.01**
	Posttest	76.50 ± 15.15		75.00 ± 15.19	
	Posttest	76.50 ± 15.15	.99	75.00 ± 15.19	.99
	Follow-up	73.17 ± 16.48		75.58 ± 13.51	
	Pretest	59.86 ± 18.17	**.01**	63.86 ± 11.83	**.01**
	Follow-up	73.17 ± 16.48		75.58 ± 13.51	

Bold indicates significant values.

CM = classical massage, CTM = connective tissue massage, PSQI = pittsburgh sleep quality index, SF-36 = short form 36.

* Post hoc Dunn test.

When the pretreatment, posttreatment, and at the end of the 6-week follow-up ODI scores were compared between the groups, no difference was found (*P* > .05) (Table 7).

### 3.4. Sleep quality

When the time-dependent changes in the PSQI were analyzed, it was found that all subcategories (without the sleep medication) provided significant improvements in the CTM group (Table 7). Since there were no CTM participants using sleep medications, the analysis could not be performed in this subcategory. However, as a result of the post hoc Dunn test, it was found that the differences in subjective sleep quality, sleep duration, sleep efficiency, and sleep disturbance were not valid (Table 8). There were statistically significant decreases in the CTM group posttreatment sleep latency and daytime sleep dysfunction compared to pretreatment (*P* = .04 and *P* = .02 respectively) (Table 8).

In the CM group, it was found that the sleep duration, sleep disturbance, and daytime sleep dysfunction subcategories provided significant improvements (Table 7). Similar to the CTM group, there were no participants using sleep medications. However, as a result of the post hoc Dunn test, the differences were found not to be valid (Table 8).

In the global score, there was a statistically significant decrease in both groups for posttreatment and at the end of the 6-week follow-up compared to the pretreatment (posttreatment *P* < .001 and at the end of the 6-week follow-up *P* = .02 in CTM group; posttreatment *P* = .01 and at the end of the 6-week follow-up *P* = .02 in CM group) (Table 8).

When the pretreatment, posttreatment and at the end of the 6-week follow-up PSQI scores were compared between the groups, no difference was found (*P* > .05) (Table 7).

### 3.5. Health-related quality of life

When the time-dependent changes in the SF-36 results were analyzed, it was found that all categories provided significant improvements in the CTM group (Table 7).

There were statistically significant improvements in the CTM group for posttreatment and at the end of the 6-week follow-up physical functioning (posttreatment *P* < .001; at the end of the 6-week follow-up *P* < .001), social functioning (posttreatment *P* < .001; at the end of the 6-week follow-up *P* = .01), role limitations due to physical health (posttreatment *P* < .001; at the end of the 6-week follow-up *P* = .01), energy/fatigue (posttreatment *P* < .001; at the end of the 6-week follow-up *P* < .001), emotional well-being (posttreatment *P* = .04; at the end of the 6-week follow-up *P* = .01), pain (posttreatment *P* < .001; at the end of the 6-week follow-up *P* = .01), general health (posttreatment *P* < .001; at the end of the 6-week follow-up *P* = .01) compared to pretreatment. In the role limitations due to emotional problems category, there was a significant increase at the at the end of the 6-week follow-up compared to at pretreatment (*P* = .04) (Table 8).

There were statistically significant improvements in the CM group for posttreatment and at the end of the 6-week follow-up physical functioning (posttreatment *P* = .01; at the end of the 6-week follow-up *P* < .001), role limitations due to physical health (posttreatment *P* = .01; at the end of the 6-week follow-up *P* = .03), pain (posttreatment *P* < .001; at the end of the 6-week follow-up *P* < .001), general health (posttreatment *P* = .01; at the end of the 6-week follow-up *P* = .01) compared to pretreatment. In the social functioning category, there was a significant increase at the end of the 6-week follow-up compared to pretreatment (*P* = .04) (Table 8). Lastly, in the energy/fatigue category, there was a significant increase at posttreatment compared to pretreatment (*P* = .04) (Table 8).

When the pretreatment, posttreatment, and at the end of the 6-week follow-up SF-36 scores were compared between the groups, the emotional well-being at the end of the 6-week follow-up score for the CTM group was significantly higher than the score for the CM group (*P* = .03) (Table 7).

## 4. Discussion

A wide variety of treatment methods have been used for CMLBP over the years.^[13]^ Among these methods, massage is at the top of the list.^[13]^ This study compared the effects of CTM and CM in patients with CMLBP. The VAS score at the end of the 2^nd^ week showed that CM decrease pain intensity significantly more than CTM. However, this effect was limited to only the 2^nd^ week. At the end of the study, both massages had similar and long-lasting effects on pain intensity. Both massage techniques can be used safely in the clinic since they did not cause any side effects during and after the study.

There are several factors that negatively affect the results of this study. Precautions were taken when planning the study to avoid negatively affecting results.

First, the “minimization” method used for randomization ensured that the participants were assigned to the groups equally by considering many parameters (age, gender, occupation). We maintained the laboratory environment under the same conditions during the evaluation and treatment to prevent negative effects that may arise from the environment. The laboratory was kept between 21°C to 25°C, not directly sun light, and the participants rested before the procedures. Finally, participants who had adapting problems to the procedures were excluded from the study.

In general, CM is used more often than CTM for the pain treatment. The mechanisms of the 2 massages are similar. Touching and pulling strokes activate subcutaneous mechanoreceptors and reveal the therapeutic effects. This results in decrease pain, regulation of autonomic response, and increase relaxation. When we look at the pain decreasing mechanisms, CTM and CM uses presynaptic and postsynaptic pathways to decrease pain.^[20,21]^

The autonomic response regulating mechanism of CTM was explained as stimulation of cutaneo-visceral reflexes with pulling strokes,^[21]^ and CM’s autonomic response regulating mechanism as similar to that of CTM. Touching during CM activates baroreceptors on blood vessels and provide autonomic changes.^[20]^ Because of the similarity of the mechanisms, it is normal that there is no difference between the groups. Future studies, comparing massages with different mechanisms will increase our knowledge of massage.

Previous studies found that CTM and CM were successful in treating low back pain and other diseases.^[12,19,23]^ Viklund et al^[34]^ conducted a similar study to the current study where the researchers examined the effect of a single-session CTM and CM in individuals with low back pain. Pain intensity was measured at baseline, directly after, and 10 minutes after treatment. The results of the study indicated that CTM may decrease pain in low back pain as effectively as CM within a short time.

In another study, patients with chronic low back pain were divided into 3 groups as CTM, placebo CTM, and control.^[23]^ The VAS was used for pain intensity assessment at rest, activity, and night, ODI was used for assessing disability, and SF-36 was used for the quality of-life assessment. At the end of the study, the results were that CTM decreased pain intensity in activity more than the sham CTM group. However, this study had a weakness. Pretreatment VAS scores were not statistically similar in the groups.

In a different study, the researchers compared the effectiveness of CM and routine treatment in subacute and chronic low back pain.^[35]^ At the end of the study, pain severity reduced in both groups, but the effect of CM was more. The study provided insights that the effect of physiotherapy will be greater when massages are included in the treatment plan. Therefore, CM and CTM were added to the treatment program while planning the current study.

Thai massage and CM were compared in a study.^[36]^ Aromatic ginger oil was used as an intermediate in CM. From the results of the study, pain decreased in both groups. In the follow-ups, the CM group had lower VASs. Ginger oil has analgesic and anti-inflammatory effects. It seems to be a suitable choice to increase the effect of the CM.

In autonomic responses, this study’s scientific results provided information to reflect on from a clinical perspective. CTM and CM produce a measurable physiological response and avoid the unwanted autonomic side effects of faintness, palpitations, and tachycardia. It was also an indication that the effects of CTM and CM lasted for a long time. There are studies that support the results of the current study and those that had different results. Similar studies reported that massage created changes in autonomic functions.^[37,38]^

In a study, a single-session CTM was applied to healthy women with different physical activity levels.^[39]^ Women aged 18-25 years were divided into CTM (n = 150) and control (n = 60) groups. Blood pressure, heart rate, oxygen saturation, skin temperature, and respiration frequency were measured immediately after the CTM. At the end of the study, respiratory frequency increased and systolic blood pressure decreased in inactive individuals, systolic blood pressure decreased in moderately active individuals, and systolic blood pressure and oxygen saturation decreased in highly active individuals. Although the study showed that CTM acutely affected autonomic functions, this study had a few weak points. At baseline, respiratory frequency, systolic and diastolic blood pressure, and body temperatures were not statistically similar between the groups. Secondly, patient distribution of the groups was not equal. Thirdly, group assignments were made by the patients’ request, not randomly.

In a different study, Holey et al^[7]^ performed a single-session CTM to eight healthy individuals. Researchers measured heart rate, blood pressure, and local and peripheral skin temperature before the CTM, immediately after, and at 15-minute intervals for 1 hour. At the end of the study, there was no change in heart rate, blood pressure, or peripheral skin temperature. But in local skin temperature, there was a temperature increase immediately after the CTM, which lasted up to 1 hour. However, there were limitations of the study. The number of participants was limited, and only females were included.

In a different study, Gholami-Motlagh et al^[40]^ examined the effects of CM on autonomic functions using a crossover research design. The researchers included healthy women in a 2-stage CM program and examined the effects on heart rate, blood pressure, respiratory rate, and body temperature. The participants were treated with back-neck-chest and leg-arm-face massages. At the end of the study, back-neck-chest massage significantly decreased systolic blood pressure, respiratory rate, and body temperature. Leg-arm-face massage significantly reduced the systolic and diastolic blood pressure, heart rate, and respiratory rate. Compared with the current study, more areas were massaged. Thus, they may have facilitated autonomic nerves in the cervical region with arm massage, cranial nerves with facial massage, and the lumbo-sacral nerves with leg massage. Therefore, greater autonomic effects may have occurred.

There are several studies that used massage therapy in disability due to CMLBP. In a study examining the effects of CTM and sham CTM on disability,^[23]^ the researchers used ODI for assessment. At the end of the study, they found that the disability decreased after treatment. When the groups were compared with each other, CTM was significantly more effective. The researchers stated that CTM was an effective treatment for disability.

In a study comparing CM and Thai massage, disability was measured using the ODI.^[36]^ Both massages decreased disability at week 6 and 15. In addition, when the groups were compared, it was stated that CM decreased disability more. The researchers stated that the reason that CM was superior was that ginger oil was used as an intermediate. The ginger oil decreased disability as well as pain intensity.

CMLBP decreases health-related quality of life. From the study, it was seen that CTM was more effective in improving quality of life. In a similar study, individuals with chronic low back pain were divided into CTM, sham CTM, and control groups.^[23]^ Their quality of life was evaluated using the SF-36. Quality of life improved in the CTM and control groups, but there was no change in the sham CTM group. When the groups were compared, there was more improvement in the CTM group.

It was found that 50% of individuals with chronic low back pain suffer from sleep problems.^[41]^ Considering the mechanism of massage techniques, improvements in pain and relaxation will increase sleep quality.^[21]^

This study has several strengths. Firstly, pain intensity was measured every 5 sessions to identify which massage reduced pain better. In this way, the change in pain intensity could be observed each week.

There are expensive and complex techniques to assess the autonomic changes. Heart rate variability, galvanic skin response, and thermal camera systems are among these techniques. Heart rate, blood pressure, and skin temperature were measured in this study. These measurements are low-cost and easy-to-use, which was the second strength side of this study.

This study also had a limitation. The study was planned as 5 sessions per week. In this design, some individuals were short of time. Setting the number of sessions to 3 or 4 per week would have made it easier to continue treatments.

## 5. Conclusion

As a result of this study, CM and CTM had almost similar effects on all parameters. They improved the participants very well, and the effects continued at the end of the 6-week follow-up. Although CTM is the first choice for modulating autonomic responses and CM for pain, we found that both massages were as effective. Considering these results, the rate of use of CM, which is the most preferred method in the clinic, will increase.

## Author contributions

**Conceptualization:** İnci Yüksel.

**Data curation:** Göktuğ Er.

**Formal analysis:** Göktuğ Er.

**Investigation:** Göktuğ Er.

**Methodology:** Göktuğ Er, İnci Yüksel.

**Project administration:** Göktuğ Er, İnci Yüksel.

**Supervision:** İnci Yüksel.

**Visualization:** Göktuğ Er.

**Writing – original draft:** Göktuğ Er.

**Writing – review & editing:** İnci Yüksel.

## References

[1] Balagué F Mannion AF Pellisé F. Non-specific low back pain. Lancet. 2012;379:482–91. 21982256 10.1016/S0140-6736(11)60610-7

[2] Van Middelkoop M Rubinstein SM Verhagen AP. Exercise therapy for chronic nonspecific low-back pain. Best Pract Res Clin Rheumatol. 2010;24:193–204. 20227641 10.1016/j.berh.2010.01.002

[3] Maher C Underwood M Buchbinder R. Non-specific low back pain. Lancet. 2017;389:736–47. 27745712 10.1016/S0140-6736(16)30970-9

[4] Larivière C Henry SM Preuss R. Structural remodeling of the lumbar multifidus, thoracolumbar fascia and lateral abdominal wall perimuscular connective tissues: a search for its potential determinants. J Anat. 2021;238:536–50. 33070313 10.1111/joa.13330 PMC7855088

[5] Tracy LM Ioannou L Baker KS. Meta-analytic evidence for decreased heart rate variability in chronic pain implicating parasympathetic nervous system dysregulation. Pain. 2016;157:7–29. 26431423 10.1097/j.pain.0000000000000360

[6] Holey LA Dixon J. Connective tissue manipulation: a review of theory and clinical evidence. J Bodyw Mov Ther. 2014;18:112–8. 24411158 10.1016/j.jbmt.2013.08.003

[7] Holey LA Dixon J Selfe J. An exploratory thermographic investigation of the effects of connective tissue massage on autonomic function. J Manipulative Physiol Ther. 2011;34:457–62. 21875520 10.1016/j.jmpt.2011.05.012

[8] Morais CA DeMonte LC Bartley EJ. Regulatory emotional self-efficacy buffers the effect of heart rate variability on functional capacity in older adults with chronic low back pain. Front Pain Res (Lausanne). 2022;3:818408. 35669039 10.3389/fpain.2022.818408 PMC9163301

[9] El-Badawy MA El Mikkawy DM. Sympathetic dysfunction in patients with chronic low back pain and failed back surgery syndrome. Clin J Pain. 2016;32:226–31. 25968450 10.1097/AJP.0000000000000250

[10] Bernstein IA Malik Q Carville S. Low back pain and sciatica: summary of NICE guidance. BMJ. 2017;356:i6748. 28062522 10.1136/bmj.i6748

[11] Deyo RA Von Korff M Duhrkoop D. Opioids for low back pain. BMJ. 2015;350:g6380. 25561513 10.1136/bmj.g6380 PMC6882374

[12] Celenay ST Kaya DO Akbayrak T. Cervical and scapulothoracic stabilization exercises with and without connective tissue massage for chronic mechanical neck pain: a prospective, randomised controlled trial. Man Ther. 2016;21:144–50. 26211422 10.1016/j.math.2015.07.003

[13] Furlan AD Giraldo M Baskwill A. Massage for low-back pain. Cochrane Database Syst Rev. 2015;2015:Cd001929. 26329399 10.1002/14651858.CD001929.pub3 PMC8734598

[14] Vlaeyen JWS Maher CG Wiech K. Low back pain. Nat Rev Dis Primers. 2018;4:52. 30546064 10.1038/s41572-018-0052-1

[15] Will JS Bury DC Miller JA. Mechanical low back pain. Am Fam Physician. 2018;98:421–8. 30252425

[16] George SZ Fritz JM Silfies SP. Interventions for the management of acute and chronic low back pain: revision 2021. J Orthop Sports Phys Ther. 2021;51:CPG1–CPG60. 10.2519/jospt.2021.0304 PMC10508241 34719942

[17] İnci Yüksel T Sezgin B Nazan T. Masaj Teknikleri. Ankara: Hipokrat Kitabevi, Pelikan Kitabevi; 2016.

[18] Kavlak E Büker N Altug F. Investigation of the effects of connective tissue mobilisation on quality of life and emotional status in healthy subjects. Afr J Tradit Complement Altern Med. 2014;11:160–5. 25371578 10.4314/ajtcam.v11i3.23 PMC4202434

[19] Bakar Y Sertel M Öztürk A. Short term effects of classic massage compared to connective tissue massage on pressure pain threshold and muscle relaxation response in women with chronic neck pain: a preliminary study. J Manipulative Physiol Ther. 2014;37:415–21. 25108749 10.1016/j.jmpt.2014.05.004

[20] Gasibat Q Suwehli W. Determining the benefits of massage mechanisms: a review of literature. Rehabil Sci. 2017;3:58–67.

[21] Sönmezer E Dökmeci F Seval MM. Connective Tissue Manipulation. In: Dökmeci F Rizk DEE, (eds). Insights Into Incontinence and the Pelvic Floor. Cham: Springer International Publishing; 2022:227–234.

[22] Celenay ST Kulunkoglu BA Yasa ME. A comparison of the effects of exercises plus connective tissue massage to exercises alone in women with fibromyalgia syndrome: a randomized controlled trial. Rheumatol Int. 2017;37:1799–806. 28840379 10.1007/s00296-017-3805-3

[23] Celenay ST Kaya DO Ucurum SG. Adding connective tissue manipulation to physiotherapy for chronic low back pain improves pain, mobility, and well-being: a randomized controlled trial. J Exerc Rehabil. 2019;15:308–15. 31111018 10.12965/jer.1836634.317 PMC6509448

[24] Yuan SLK Matsutani LA Marques AP. Effectiveness of different styles of massage therapy in fibromyalgia: a systematic review and meta-analysis. Man Ther. 2015;20:257–64. 25457196 10.1016/j.math.2014.09.003

[25] Cabak A Podgórski J Rekowski W. Application of thermal imaging in the assessment of skin warming after classic massage and rubber cup massage. Compl Alter Med Sci. 2013;1:55–9.

[26] Chiarotto A Maxwell LJ Ostelo RW. Measurement properties of visual analogue scale, numeric rating scale, and pain severity subscale of the brief pain inventory in patients with low back pain: a systematic review. J Pain. 2019;20:245–63. 30099210 10.1016/j.jpain.2018.07.009

[27] Vischer AS Burkard T. Principles of blood pressure measurement – current techniques, office vs ambulatory blood pressure measurement. In: Islam MS, ed. Hypertension: From Basic Research to Clinical Practice. Advances in Experimental Medicine and Biology, vol 956. Cham: Springer; 2016. doi:10.1007/5584_2016_49. 10.1007/5584_2016_49 27417699

[28] Muntner P Shimbo D Carey RM. Measurement of blood pressure in humans: a scientific statement from the American Heart Association. Hypertension. 2019;73:e35–66. 30827125 10.1161/HYP.0000000000000087 PMC11409525

[29] Gatt A Formosa C Cassar K. Thermographic patterns of the upper and lower limbs: baseline data. Int J Vasc Med. 2015;2015:1–9. 10.1155/2015/831369 PMC4310239 25648145

[30] Lahiri B Bagavathiappan S Jayakumar T. Medical applications of infrared thermography: a review. Infrared Phys Technol. 2012;55:221–35. 32288544 10.1016/j.infrared.2012.03.007 PMC7110787

[31] Chiarotto A Maxwell LJ Terwee CB. Roland-Morris disability questionnaire and oswestry disability index: which has better measurement properties for measuring physical functioning in nonspecific low back pain? Systematic review and meta-analysis. Phys Ther. 2016;96:1620–37. 27081203 10.2522/ptj.20150420

[32] Chapman JR Norvell DC Hermsmeyer JT. Evaluating common outcomes for measuring treatment success for chronic low back pain. Spine. 2011;36:S54–68. 21952190 10.1097/BRS.0b013e31822ef74d

[33] Marin R Cyhan T Miklos W. Sleep disturbance in patients with chronic low back pain. Am J Phys Med Rehabil. 2006;85:430–5. 16628150 10.1097/01.phm.0000214259.06380.79

[34] Viklund P Hustad T Danielsson F. A comparison of the effects of connective tissue massage and classical massage on low back pain;A randomized controlled trial. J Bodyw Mov Ther. 2015;19:672.

[35] Kamali F Panahi F Ebrahimi S. Comparison between massage and routine physical therapy in women with sub acute and chronic nonspecific low back pain. J Back Musculoskelet Rehabil. 2014;27:475–80. 24867893 10.3233/BMR-140468

[36] Sritoomma N Moyle W Cooke M. The effectiveness of Swedish massage with aromatic ginger oil in treating chronic low back pain in older adults: a randomized controlled trial. Complement Ther Med. 2014;22:26–33. 24559813 10.1016/j.ctim.2013.11.002

[37] Diego MA Field T. Moderate pressure massage elicits a parasympathetic nervous system response. Int J Neurosci. 2009;119:630–8. 19283590 10.1080/00207450802329605

[38] Lee Y-H Park BNR Kim SH. The effects of heat and massage application on autonomic nervous system. Yonsei Med J. 2011;52:982–9. 22028164 10.3349/ymj.2011.52.6.982 PMC3220246

[39] Akbaş E Ünver B Erdem EU. Acute effects of connective tissue manipulation on autonomic function in healthy young women. Complement Med Res. 2019;26:250–7. 30933948 10.1159/000497618

[40] Gholami-Motlagh F Jouzi M Soleymani B. Comparing the effects of two Swedish massage techniques on the vital signs and anxiety of healthy women. Iran J Nurs Midwifery Res. 2016;21:402–9. 27563325 10.4103/1735-9066.185584 PMC4979265

[41] Kelly GA Blake C Power CK. The association between chronic low back pain and sleep: a systematic review. Clin J Pain. 2011;27:169–81. 20842008 10.1097/AJP.0b013e3181f3bdd5

